# Effectiveness of the back school and mckenzie techniques in patients with chronic non-specific low back pain: a protocol of a randomised controlled trial

**DOI:** 10.1186/1471-2474-12-179

**Published:** 2011-08-05

**Authors:** Alessandra N Garcia, Francine LB Gondo, Renata A Costa, Fábio N Cyrillo, Tatiane M Silva, Luciola CM Costa, Leonardo OP Costa

**Affiliations:** 1 Masters Program in Physical Therapy, Universidade Cidade de São Paulo, Brazil; 2 School of Physical Therapy, Universidade Cidade de São Paulo, Brazil; 3 Musculoskeletal Division, The George Institute for Global Health, Sydney, Australia

## Abstract

**Background:**

Chronic low back pain is a highly prevalent condition, which is associated with high direct and indirect costs to the society. Although this condition is highly prevalent, it is still extremely difficult to treat. Two potentially useful treatments for patients with chronic low back pain are called the *McKenzie *and *Back School *treatment programs. These programs have good biological plausibility, are widely available and have a modest cost. Although these treatments are already available for patients, the evidence that supports their use is largely based on low quality methodological studies. Therefore, a high-quality randomised controlled trial is required to compare, for the first time, the effectiveness of these treatments in patients with chronic low back pain.

**Methods/design:**

One hundred and forty-eight patients will be randomly allocated to a four-week treatment program based upon the *McKenzie *or *Back School *principles. Clinical outcomes (pain intensity, disability, quality of life, and trunk flexion range of motion) will be obtained at follow-up appointments at 1, 3 and 6 months after randomisation. The data will be collected by an assessor who will be blinded to the group allocation.

**Discussion:**

This will be the first study aimed to compare the *McKenzie *and *Back School *approaches in patients with chronic low back pain. The results of this trial may help in the decision-making process of allied health providers for the treatment of chronic low back pain and reduce the health-related costs of this condition.

**Trial Registration:**

ACTRN12610000435088

## Background

Low back pain is an important health problem with significant consequences from a socio-economic point of view and is associated with high costs, work absenteeism and disability. Prevalence estimates of low back pain vary considerably depending on the population being studied as well as with regards to the definition of a low back pain episode. The only systematic review on this topic estimated that the point prevalence of low back pain ranges from 12 to 33%, the one-year prevalence of low back pain ranges from 22 to 65%, and the lifetime prevalence of low back pain ranges from 11 to 84%[1].

Given the high prevalence of this condition, low back pain is considered to be an important public health problem in many countries, including the United States [2], Australia [3], and European countries [4]. The direct and indirect costs that are associated with low back pain are immense; for example, the costs associated with low back pain in Australia are more than 1 billion Australian dollars, while in the United States of America, the annual costs associated with low back pain were estimated in 50 billion American dollars [2,5]. Regardless of the observed differences in the costs to health systems in different countries, there is no doubt that low back pain represents an important economic problem worldwide.

Estimates of the prognosis of chronic low back pain are based on a limited number of studies. The largest prospective inception cohort that investigated the prognosis of chronic low back pain was conducted in Australia [6]. This study followed 406 patients with a recent episode of chronic low back pain for 12 months and observed that 35% of the patients completely recovered by 9 months and that only 41% recovered by 12 months. Clearly, the probability of recovery is considerably lower for chronic patients compared to patients with acute low back pain, making it necessary to develop better treatments for patients with chronic low back pain.

A range of therapeutic possibilities are available for patients with chronic low back pain. These treatments include educational programs [7], cognitive behavioural therapy [8], medication [9-11], electrotherapy and thermotherapy [12-14], manual therapy [15], and exercise [16]. The majority of these treatments are recommended by the *European Guidelines for the Management of Chronic Low Back Pain*[4], where the options considered to have the most benefit for patients are exercise combined with education programs and exercise programs that follow cognitive behavioural therapy principles. There is no evidence that suggests that any other type of treatment is better than programs that use exercise as the basis for the treatment of low back pain [4]. However, the literature is not clear with regards to which exercise programs are most effective for patients with chronic low back pain; therefore, better randomised controlled trials are necessary to clarify these questions. This need is emphasised by the *European Guidelines*, which suggest "that the effectiveness of specific exercise programs needs to be evaluated, specially programs that are commonly utilised but which have been inadequately researched (p. S196)"[4].

The *Back School *(a group-based treatment approach) and the *McKenzie *(an individually-based treatment approach) methods are promising therapeutic options [17,18] that use exercise for the treatment of low back pain in addition to delivering theoretical information in order to educate patients about their condition, so that patients are better able to understand their condition and how to change their behaviour towards an episode of low back pain.

The *Back School *method was developed in 1969 in Sweden by Mariane Zachrisson Forssel, with the goal of preventing and avoiding recurrent episodes of low back pain. The program is composed of 4 sessions lasting approximately 45 minutes, being each session organised by theoretical components, which include: anatomy and spinal biomechanics; epidemiology; physiopathology of the most frequent back disorders; posture; ergonomics; common treatment modalities and a practical component (exercises for the maintenance of a "healthy back")[18].

In 1981, Robin McKenzie proposed a classification system and individualised treatment regimen for low back pain called "diagnostic and mechanical therapy" or, simply, the *McKenzie*[19] method. The *McKenzie *method consists of three steps: evaluation, treatment, and prevention. The evaluation step is conducted using repeated movements and sustained positions where the symptoms in the lower back and lower limbs are classified into three subgroups: 1) derangement syndrome, 2) dysfunction syndrome, and 3) posture syndrome [19]. The choice of appropriate exercises in the *McKenzie *method is based upon the direction (flexion, extension, or lateral shift of the spine) that can lead to the following possible responses: pain reduction, "centralisation of symptoms" (i.e., symptoms migrating towards the middle line of the body), and the complete recovery of pain. The prevention step consists of educating and encouraging the patient to exercise regularly and self-care [17].

Although both methods have been previously investigated, there have been criticisms regarding the methodological quality of these popular treatment programs for patients with chronic low back pain [17,20]. Examples of these problems are: 1) lack of assessor blinding 2) small sample sizes, 3) inappropriate statistical analysis, 4) problems with the random allocation of participants, and 5) loss of follow up greater than 15%. The occurrence of one or more of the problems mentioned above introduces bias that compromises the internal and external validity of the study. Moreover, no study has directly compared the results of each technique on this condition. Therefore, the objective of this study is to compare, for the first time, the effects of the *McKenzie *and *Back School *techniques in patients with chronic low back pain. Our goal is to conduct a robust, randomised controlled trial from both a methodological point of view as well as in terms of generalizability.

## Methods/Design

### Approval and registration of the study

The study commenced recruitment in May 2011 at the outpatient Physiotherapy Clinic of the Universidade Cidade de São Paulo (UNICID), in São Paulo/Brazil. The study design, procedures and informed consent were approved by the Ethics Committee in Research of UNICID (research protocol number 1349394) and was prospectively registered [21] in the *Australian and New Zealand Clinical Trials Registry *(registration number ACTRN12610000435088).

### Eligibility criteria

We will recruit patients presenting with low back pain for at least three months and aged between 18 and 80 years. Participants with any contraindication to physical exercise [22] will be excluded from the study based on the guidelines of the *American College of Sports Medicine*. Patients with serious spinal pathology (including fractures, tumours, and inflammatory diseases), nerve root compromise, cardio respiratory illnesses, and pregnancy will be also excluded.

### Procedures

Potential participants in the study will be welcomed by the study assessor who will determine which patients will or will not participate in the study, in accordance with the previously described eligibility criteria. The patients will receive information about the study and the criteria for study eligibility. If the patient is considered eligible, the assessor will collect the baseline data prior to randomisation. The assessor will be blinded with regards to the allocation of patients into the treatment groups. The primary outcomes of our study are pain intensity and disability at 4-weeks after randomisation. The secondary outcomes are range of motion of trunk flexion at 4-weeks, pain intensity and disability in evaluations at 3 and 6 months after randomisation, and quality of life in the evaluations at 4 weeks, 3 months and 6 months after randomisation. The study assessor will be unaware of the treatment that the patients received. Given the nature of the study, it will not be possible for the therapist or the patients to be blinded, which is expected in studies that compare the effectiveness of complex interventions such as exercise therapy.

### Randomisation procedures

**Figure 1:**
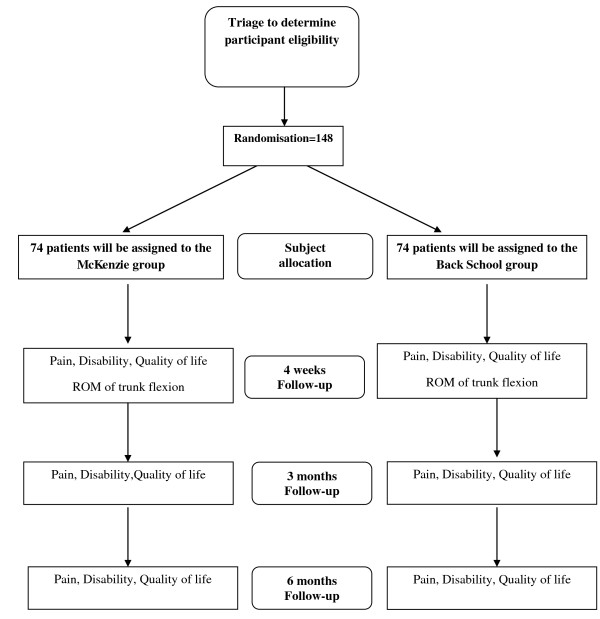
**Flow diagram of the study**.

Immediately after the baseline evaluation, patients will be sent to the physiotherapist responsible for the interventions. Before initiating the treatment, the patients will be randomly allocated into the two treatment arms *(Back School *or *McKenzie*) through a computer-generated randomisation schedule that was performed by one of the investigators who is not involved in the recruitment or treatment of the patients. The allocation of subjects will be concealed by using consecutive numbered, sealed and opaque envelopes [23]. Before initiating the intervention, the therapist responsible for the treatment will open the envelope in front of the patient and will inform the patient of the treatment technique corresponding to the number on his/her envelope. For a better visualisation of the study outline, see Figure 1.

### Outcomes

To evaluate the participants, four instruments will be used: 1) *pain intensity *will be measured by the *Pain Numerical Rating Scale (NRS)*; 2) *disability associated with back pain *will be measured by the *Roland Morris Disability Questionnaire; *3) a *Fleximeter^© ^*will be used for measuring the *range of movement (ROM) of the trunk flexion*, and 4) the *WHOQOL-Bref *[24] will be used to measure *quality of life*. A detailed description of each of these instruments is described as it follows.

#### Pain Numerical Rating Scale (NRS)

The *Pain Numerical Rating Scale (NRS) *measures the levels of pain intensity perceived by the patient using an 11-point scale that ranges from 0 to 10, where 0 is classified as "no pain" and 10 is classified as "worst possible pain"[25,26]. The participants will be asked to rate their levels of pain intensity based upon the last 7 days. This outcome will be measured in all time-points (i.e., baseline, 4 weeks, 3 months, and 6 months after randomisation).

#### Roland Morris Disability Questionnaire

The *Roland Morris Disability Questionnaire *is an instrument that is widely used in research and clinical practice for the measurement of disability associated with low back pain and has been translated, cross-culturally adapted and clinimetrically tested for the Brazilian population [25-27]. It is composed by 24 items that describe daily situations that patients may have difficulty to perform because of their low back pain problem. The greater the number of items selected, the greater the disability. Participants will be asked to indicate the items that describe them on the day of the assessment. This outcome will be measured in all time-points (i.e., baseline, 4 weeks, 3 months, and 6 months after randomisation).

### Fleximeter

The Fleximeter (*Fleximeter^©^*) is a measurement tool used for measuring flexibility and range of motion through an angular scale based on the mechanism of gravitational action. As it is an instrument that does not have articular vertices, but rather has a velcro that is placed at the articulation of interest which allows a number of diverse joint movements to be isolated, guaranteeing a measurement precision. The range of back flexion movement will be measured with the patient in orthostatic position with their knees extended and arms crossed across the thorax. The fleximeter will be positioned laterally in the thoracic region at breast height with the subject facing the assessor. The patient will be asked to bend his/her trunk to the maximum range possible. For logistical reasons (given a larger potential for missing data compared with the self-report outcome measures that can be measured over the phone, email or letter), we will measure ROM only at the baseline and at the 4 weeks follow-up.

### WHOQOL-Bref Questionnaire

The *WHOQOL-Bref *[24] quality of life questionnaire corresponds to a short version of the WHOQOL-100 that maintains the original clinimetric characteristics and is adapted for the Brazilian-Portuguese language. The *WHOQOL-Bref *is composed of 26 questions: two general quality of life questions, and 24 questions that represent each of the domains that the original instrument contains. It evaluates four quality of life factors: physical, psychological, social and environmental. Participants will be asked to respond to the questions with regards to the last 2 weeks. This outcome will be measured in all time-points (i.e., baseline, 4 weeks, 3 months, and 6 months after randomisation).

### Interventions

As the *Back School *method requires four treatment sessions [18], the same number of sessions will be used for the *McKenzie *method group in order to standardise the total treatment time for both groups. Table 1 displays a summarised description of the treatments that will be provided in this study.

**Table 1:** Summarised description of the treatment programs

	*McKenzie *Method	*Back School Method*
**1^st ^week**	-Presentation of the proposed methods, history, and general information about the Mckenzie method; -Completion of the exercises after initial evaluation results and indication of preference: flexion, extension, or lateral shift of the spine; -Educational component: basic information about the lower back and its structure; mechanical pain; how and why to do exercises; and types of responses that can occur in response to the exercise program; -Guidance on completing the exercises at home.	-Presentation of the proposed methods, history, and general information about the Back School method; -Anatomy and biomechanical concepts of the spine; -Epidemiology; -Muscle function and its influence on the spine; -Physiopathology of the main disorders that negatively affect the back; -Principal treatment modalities.

**2^nd ^week**	-Progression of the exercises defined after the 1^st ^session and progression towards other positions in line with the responses of the patient. -Educational component: basic information about the most common causes of low back pain, emphasising posture when seated for a prolonged time; practice on finding the correct seated position and maintenance of back lordosis in this position. -Guidance on continuing exercises at home.	-Variation of the mechanical forces in different movements of the back; -Relaxation posture; -Guidance on positions when seated or standing; -Instruction on breathing exercises, kinaesthetic training, stretching of the lower back, quadriceps, and hamstrings; -Guidance on completing exercises at home once a day.

**3^rd ^week**	-Progression of the exercises defined after the 2^nd ^session and progression towards other positions in line with the responses of the patient. -Educational component: basic information about the most common causes of low back pain; emphasising work on bending positions; standing up; relaxing after vigorous activity; remaining in standing position for prolonged periods; lying down; and resting, coughing, and sneezing. - Guidance on continuing the exercises at home.	-Observation of the exercises completed at home; -Instruction on exercises for abdominal muscular strength; -Practical application of techniques for joint protection; - Guidance on how to perform the exercises at home once a day.

**4^th ^week**	-Progression of the exercises defined after the 3^rd ^session and progression towards other positions in line with the responses of the patient. -Educational component: review of the most important points since the first week.	-Practical application of all the exercises and learned techniques.

#### McKenzie Group

Participants in the *McKenzie *group will receive four individual sessions, once per week, lasting an average of 45 minutes to an hour each. Treatment will be provided in accordance with the direction of the preference of movement, or rather, flexion, extension or lateral shift of the spine [19]. The program will be divided based on theoretical and practical information (Tables 1 and 2).

**Table 2:** Description of the exercises for the *McKenzie *group

Exercise	Position	Series
Trunk Flexion	**Lying down: **prone position with knees and hips flexed and feet supported on the plinth. The patient is instructed to raise the knees towards the chest, applying extra pressure with the hands towards the knees. **Seated: **seated in a chair with the knees and hips at 90 degrees, the patient shifts the forward, until the head is between the knees and the hands are as close as possible to the floor. For the most effective effect, the patient can hold the ankles bringing the trunk even closer to the front. **Standing: **with the feet placed shoulder-width apart, the patient places his hands on the front part of the thighs gliding them as much as possible in the direction of the floor keeping the knees extended.	3 sets of 10 repetitions Could be performed sequentially with a small break between them or divided at distinct times of day in accordance with the responses of the patient.

Trunk Extension	**Lying down: **patient begins in prone position with the palms of the hands facing down below the shoulders. Patient extends the elbows, elevating the upper part of the body, while the pelvis and the thighs remain relaxed. **Standing: **with the feet placed shoulder-width apart and the hands placed at the base of the low back with fingers pointed towards the floor, incline the trunk backwards for as long as possible, keeping the head relaxed.	3 sets of 10 repetitions Could be performed sequentially with a small break between them or divided at distinct times of day in accordance with the responses of the patient.

Lateral Shift	**Standing with upper arm support: **with the feet placed shoulder-width apart and the upper arm supine at 90° of elbow flexion in contact with the lateral trunk toward the shifting side, using the other hand, shift the pelvis to the side supported by the upper arm. **Standing with wall support: **with the feet placed shoulder-width apart, support one of the upper arms on the wall and use the other hand to shift the pelvis in the direction of the wall.	3 sets of 10 repetitions Could be performed sequentially with a small break between them or divided at distinct times of day in accordance with the responses of the patient.

#### Back School Group

The participants in the *Back School *group will receive four treatment sessions, once per week, lasting an average of 45 minutes to one hour each. As the participants will commence the treatments immediately after randomisation; the first session of the *Back School *method will be conducted individually. The remaining sessions will be conducted as group sessions. The program will be divided based on theoretical and practical information (Tables 1 and 3).

**Table 3:** Description of the exercises for the *Back School *group

Exercise	Position	Sets/Duration
Diaphragmatic breathing	Seated, inhale slowly and deeply through the nose, elevating the abdomen. Breathe air out the through the mouth, raising the navel in the direction of the back.	1 set of 10 repetitions

Stretching of the erector spinae muscles	Supine position with flexed knees and supported feet. Bring first one knee and then the other toward the thorax, join the hands across the thighs, and push them in the direction of the thorax.	30 seconds Repeat 10 times

Stretching of the posterior lower limbs muscles	Supine position with one of the legs supported on the mattress and the other flexed approximately 90° at the hip and knees extended, maintained with help from a bed sheet.	30 seconds Repeat 10 times

Stretching of the anterior hip muscles	Lying down in lateral decubitus over the limb that will be stretched with the hip in a neutral position and knees flexed. Raise the heel of the leg underneath in the direction of the gluteus, keeping the back aligned. Contra lateral member in triple flexion of 90° and the internal side of the knee supported by the mattress.	30 seconds Repeat 10 times

Kinaesthetic training	Seated, move the pelvis, making a front and back pelvic inclination at a comfortable range.	1 set of 10 repetitions

Strengthening of the abdominal musculature	a) Prone position with two feet supported on the mattress, upon exhaling raise head, shoulders, and thorax, with arms placed at the side of the body, maintaining the alignment of the head with the cervical spine. Contracting the transverse abdominis, pelvic floor and paravertebral muscles. b) Prone position with head supported on the mattress, hands at the side of the body. With an extension of the knee of one leg, raise it in the direction of the mattress surface, while the other remains in triple flexion in contact with the trunk. Before the extended leg touches the mattress, alternate the movement bringing it in flexion, while the other is stretched. The leg extension should be performed while exhaling, maintaining transverse abdominis, paravertebral, and pelvic floor muscles contracted.	a) 1 set of 10 repetitions b) 1 set of 10 repetitions for each leg.

### Statistical analysis

#### Sample size calculation

The sample size calculation for this study was performed to detect a between-group difference of 1 point in the outcome pain intensity as measured by the Portuguese version of the *Pain Numerical Rating Scale*[25] (with an estimated standard deviation of 1.84 points) and of 4 points in the disability outcome measured by the Roland Morris Disability Questionnaire [26] (with an estimated standard deviation of 4.9 points) with an statistical power of 80%, an alpha of 5%, and a possible loss of follow up of 15%. Given these parameters, 74 patients per group or a total of 148 participants will be necessary.

#### Treatment effectiveness analysis

All of the statistical procedures will be conducted on a intention-to-treat basis [28]. In our primary analysis, we will use linear mixed models model to test for the effect of treatment on outcomes at 1, 3 and 6 months follow-up. A treatment effect size will be calculated for each of the follow-up time points and, if there is a statistically significant treatment effect at any time point. The software packages *SPSS 19 *and *SigmaPlot 10 *will be used for this analysis.

## Discussion

In this study, we present the rationale and design for a randomised controlled trial comparing the effects of the *McKenzie *and *Back School *methods in patients with chronic low back pain. The results of this study will be published once the study is concluded.

## Declaration of Competing interests

The authors declare that they have no competing interests.

## Authors' contributions

ANG, FLBG, RAC, FNC and LOPC were responsible for designing the study. TMS and LCMC will act as the study coordinators. All authors read and approved the final manuscript.

## Pre-publication history

The pre-publication history for this paper can be accessed here:

http://www.biomedcentral.com/1471-2474/12/179/prepub
